# Supplementary material for: KegAlign: optimizing pairwise alignments with diagonal partitioning
Source: Genome Biol. 2025 Nov 17;26:389. doi: 10.1186/s13059-025-03830-0 (PMC12621372; doi:10.1186/s13059-025-03830-0)
Supplement: Supplementary file 1 — Additional file 1: Figure S1. Human – Primate chr1 alignment. Each chromosome has ~ 250 million nucleotides. Each segment file contains HSPs from a single chunk. Each box indicates a 10 × 10Mbp chunk. Some chunks can have very few alignments as shown in the blue box, while others can have significantly more, as shown in the red box. Figure S2. KegAlign workflow available at https://usegalaxy.org/u/cartman/w/imported-basic-pairwise-alignment [33]. [file 13059_2025_3830_MOESM1_ESM.pdf]

Supplemental Figures

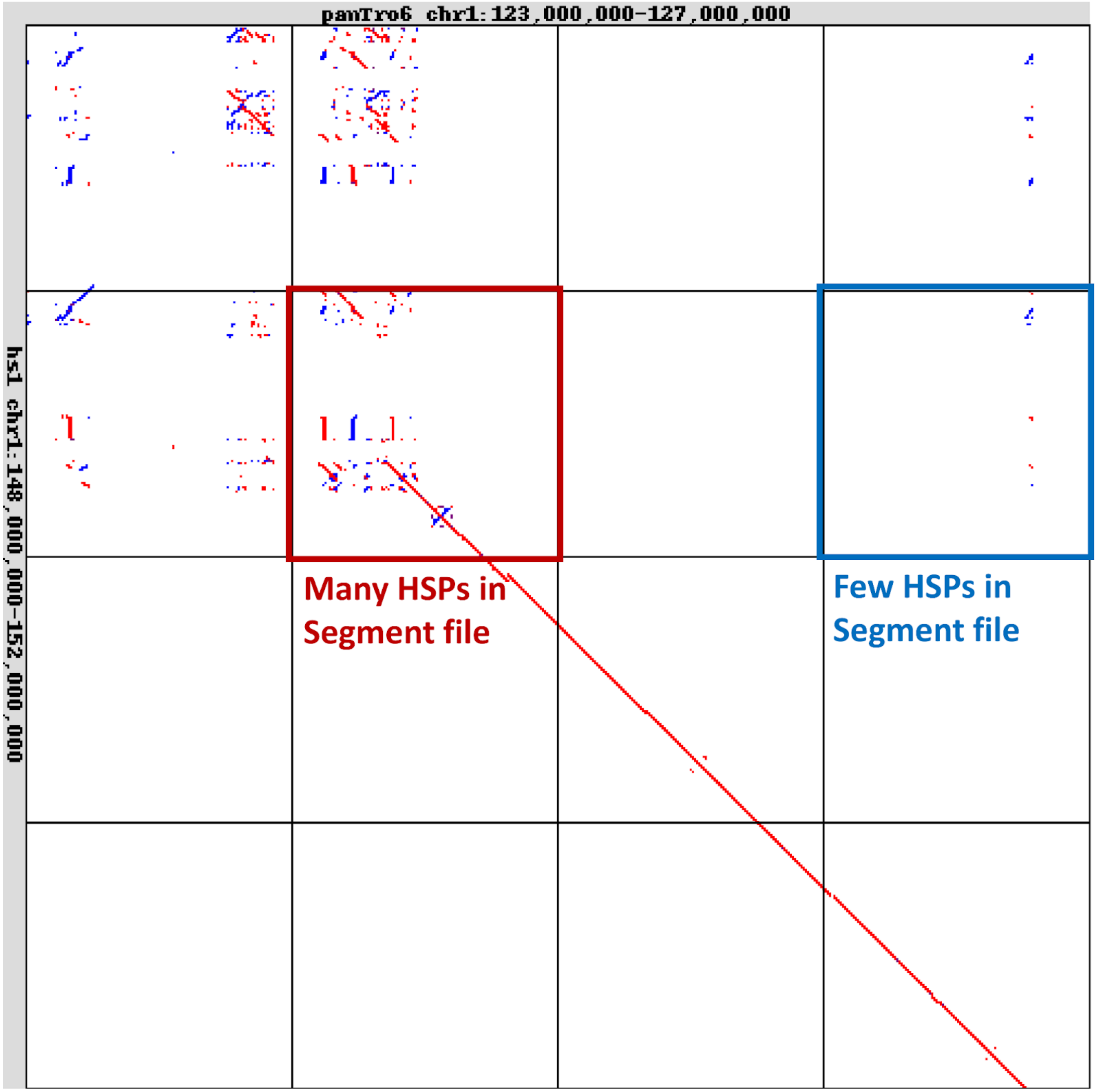

Figure S1. Human – Primate chr1 alignment. Each chromosome has ~250 million nucleotides. Each segment file contains HSPs from a single chunk. Each box indicates a 10x10Mbp chunk. Some chunks can have very few alignments as shown in the blue box, while others can have significantly more, as shown in the red box.

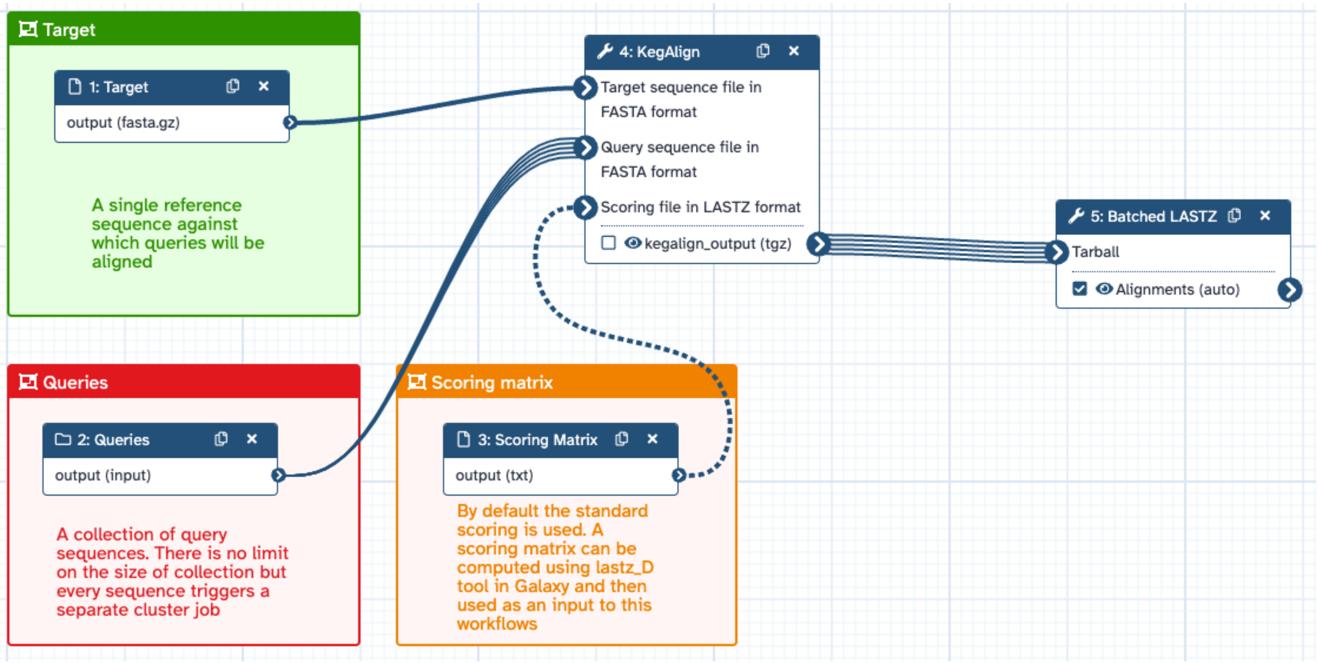

Figure S2. KegAlign workflow available at https://usegalaxy.org/u/cartman/w/imported-basic-pairwise-alignment [33]
